# Whole-genome sequencing based on formalin-fixed paraffin-embedded endomyocardial biopsies for genetic studies on outcomes after heart transplantation

**DOI:** 10.1371/journal.pone.0217747

**Published:** 2019-06-05

**Authors:** Gustav Zar, J. Gustav Smith, Maya Landenhed Smith, Bodil Andersson, Johan Nilsson

**Affiliations:** 1 Department of Clinical Sciences Lund, Cardiothoracic Surgery, Lund University and Skane University Hospital, Lund, Sweden; 2 Department of Clinical Sciences, Cardiology, Lund University and Skane University Hospital, Lund, Sweden; 3 Wallenberg Center for Molecular Medicine, Lund University, Lund, Sweden; 4 Department of Clinical Sciences Lund, Surgery, Lund University and Skane University Hospital, Lund, Sweden; University of Toledo, UNITED STATES

## Abstract

**Background:**

Whole-genome sequencing (WGS) of heart transplant recipient- and donor-derived cardiac biopsies may facilitate organ matching, graft failure prediction, and immunotolerance research. The objective of this study was to determine the feasibility of WGS based on formalin-fixed paraffin-embedded endomyocardial biopsies.

**Methods and results:**

The study included serial donor- and recipient samples from patients who had undergone heart transplantation at Skane University Hospital, Lund, Sweden, between 1988 and 2009. DNA extraction and WGS were conducted. Additional WGS sequencing quality metrics and coverage were obtained with the *Genome Analysis Toolkit (GATK)*.

455 endomyocardial samples from 37 heart transplant recipients were acquired from routine rejection monitoring and stored as formalin-fixed paraffin-embedded samples. They were analyzed after 3–26 years of storage. DNA was extracted from 114 samples and WGS was run on 85 samples. DNA extraction yielded 313 ng (IQR 96–601) for all samples. A coverage of 11.3x (IQR 9.0–15.9) was recorded for all WGS samples. Three samples stored for > 25 years yielded a coverage of > 25x. Data were generated for 1.7 billion reads per sample (IQR 1.4–2.7). A Transition/Transversion (TiTv) ratio of 2.09 ± 0.05 was calculated for all WGS samples. No associations were found among storage time, DNA yield, or sequencing quality metrics.

**Conclusions:**

The present study demonstrated the feasibility of whole-genome sequencing based on endomyocardial biopsies. This process could enable large-scale retrospective genomic studies using stored histopathological samples.

## Introduction

Post-heart transplantation survival has improved over the last three decades. Median survival now exceeds 12 years [1]. Management of long-term complications such as malignancy underscores the importance of customizing immunosuppression in transplant patients [2]. Graft failure remains the most common cause of death and is often the result of immune-mediated rejection [3].

Whole-genome sequencing (WGS) technologies comprehensively characterizes human leukocyte antigen (HLA) genes located on chromosome 6 and other genomic regions determining immune tolerance. Elucidation of the genetic predisposition to rejection may help tailor immunosuppressive therapy and lower the incidence of malignancies in closely matched patients.

Most next-generation sequencing (NGS) studies have used DNA isolated from whole blood or fresh frozen tissues. However, experiments involving formalin-fixed paraffin-embedded (FFPE) samples have demonstrated that FFPE may be valuable as a DNA source for NGS. These studies have indicated a significant influence of storage time on sequencing quality [4]. Targeted NGS on cardiac biopsies using sequencing panels demonstrated no significant differences between FFPE- and blood samples and adequate sequencing scores for older samples [5].

The endomyocardial biopsies (EMB) obtained from routine rejection monitoring after heart transplantation are FFPE samples. The feasibility of FFPE in WGS of EMB and the effects of storage time on these tissue specimens must be determined. If it is validated, this study design would support large-scale retrospective genomic studies on EMBs derived from FFPE samples of numerous heart transplantations.

The objectives of this study were to determine whether DNA obtained from archived EMB stored as FFPE suffices for WGS and to assess the effects of storage time on sequencing quality.

## Results

### Study population characteristics

A total of 455 FFPE samples were collected from 37 transplanted patients and used in DNA extraction and WGS. The mean recipient age was 45 ± 12 years with a range of 13–59 years. The mean survival after transplantation was 11.4 ± 6.5 years. The mean donor age was 36 ± 14 years with a range of 13–64 years. Most recipients were male (59%, n = 22) as were most donors (54%, n = 19). Heart failure was caused by non-ischemic cardiomyopathy in 62% of the recipients (n = 23), ischemic cardiomyopathy in 32% of the recipients (n = 12), and cardiac sarcoidosis and tumor cordis in the remaining 5% (n = 2).

### Quantity and quality of DNA extracted

**Fig 1:**
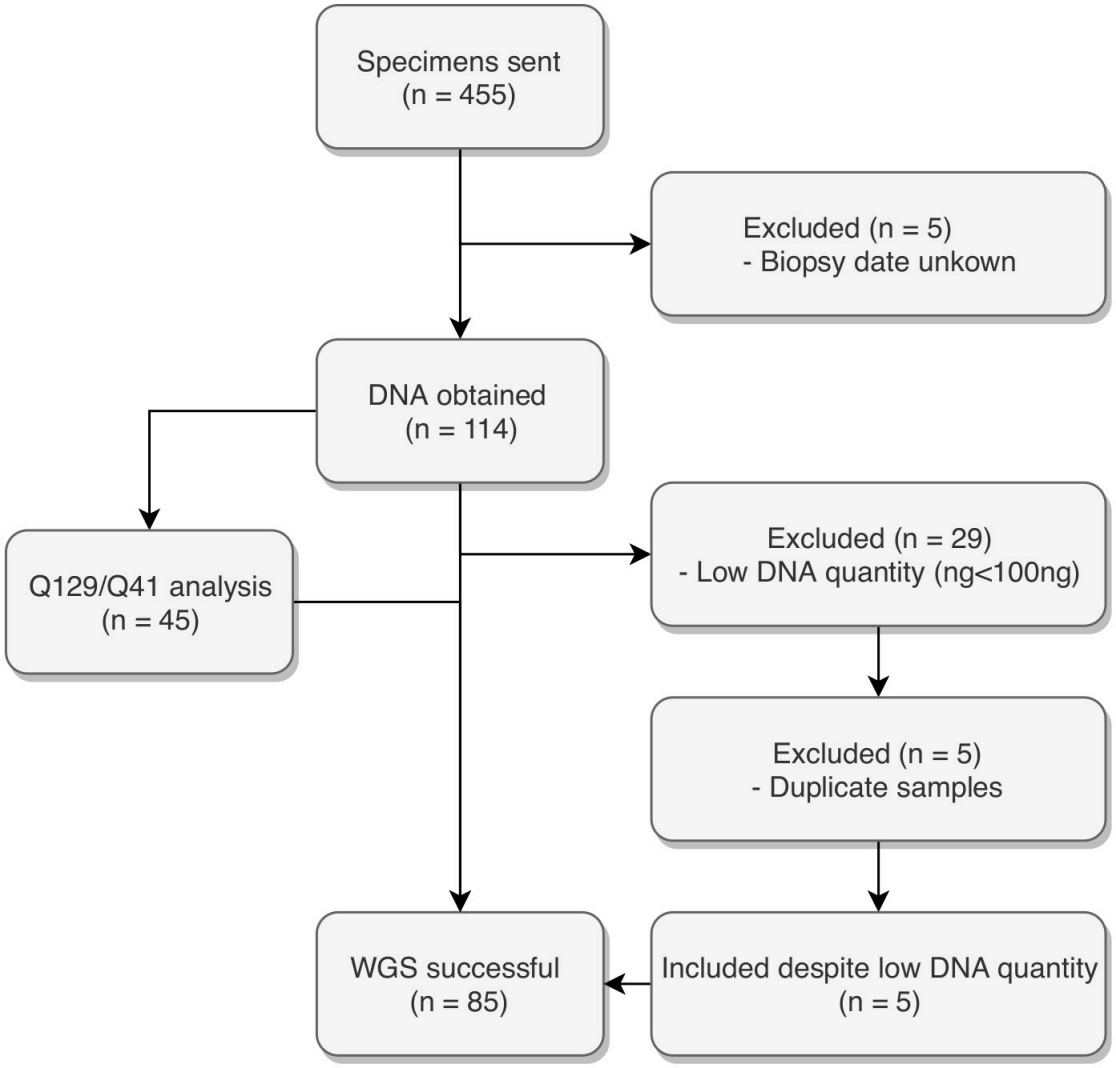
Patient flow.

The DNA extraction from the 455 FFPE samples generated 119 unique samples. Of these, 114 had known exact storage times (Fig 1) and their median DNA extraction was 313 ng (IQR 96–601) (Table 1). The median KAPA QC score (Q129/Q41 ratio, n = 45) was 0.003 (IQR 0.0007–0.0178).

**Table 1:** DNA quantity (nanogram) following DNA extraction according to sample storage time.

Variable	All n = 114	0–10 y n = 13	11–15 y n = 18	16–20 y n = 34	21–30 y n = 49	Min	Max
DNA yield	313 (96–601)	481 (186–903)	326 (40–601)	442 (259–747)	162 (66–350)	7	3,388

Data are expressed as medians and interquartile ranges.

### Whole-genome sequencing analysis

The median coverage was 11.3x (IQR 9.0–15.9) for all samples (Table 2). We observed no significant difference in coverage between Illumina HiSeq 2000 (n = 10) and Illumina HiSeq X (n = 75; *P* = 0.2469). The median percentage of callable bases at Phred score ≥ Q20 was 77.0 (IQR 70.8–82.1). The median percentages of genome covered at 20x, 10x, and 0x for all WGS samples were 15.8 (IQR 12.4–29.4), 40.9 (IQR 25.3–58.4), and 2.9 (IQR 1.9–4.0), respectively. We recorded a mean library size of 1.7 gigabases (IQR 1.4–2.7) for all WGS samples and a Transition/Transversion (TiTv) ratio of 2.09 ± 0.05 (Table 2). We compared the TiTv ratios for our FFPE samples with those from the human genome sample NA12878 which yielded a TiTv ratio of 2.13. Maximum coverage was 55x recorded for a sample with a storage time of 11 years. This sample had 97.2% of the genome covered at 20x and 1.1% of the genome covered at 0x. Three samples with storage times > 25 years yielded coverages > 25x. The sample with the least coverage had a DNA yield of 162 ng and a KAPA QC score of 0.01.

**Table 2:** Whole-genome sequencing quality metrics according to sample storage time.

Variable	All n = 85	0–10 y n = 11	11–15 y n = 12	16–20 y n = 28	21–30 y n = 34	Min	Max
Coverage (x)	11.3 (9.0–15.9)	12.2 (10.7–18.2)	10.9 (9.6–14.6)	10.4 (9.2–17.7)	11.2 (8.4–15.4)	3.8	55.5
% Covered 20x	15.8 (12.4–29.4)	16.0 (11.2–29.7)	13.6 (11.3–22.0)	14.9 (13.9–31.2)	17.3 (11.1–29.4)	0.8	97.2
% Covered 10x	40.9 (25.3–58.4)	54.7 (43.4–61.5)	42.0 (28.4–60.9)	38.2 (26.3–63.2)	36.0 (20.3–53.2)	9.3	97.9
% Covered 0x	2.9 (1.9–4.0)	2.2 (1.9–3.0)	2.7 (2.0–3.7)	2.9 (1.9–3.5)	3.4 (2.2–4.7)	1.1	26.2
% Q20	77.0 (70.8–82.1)	80.8 (75.7–84.0)	79.4 (73.4–83.1)	76.8 (73.0–84.0)	74.2 (67.7–79.8)	40.5	93.9
Library size	1.7 (1.4–2.7)	2.8 (1.7–3.2)	1.7 (1.4–2.0)	1.7 (1.5–3.0)	1.6 (1.3–2.4)	0.87	8.61
Ti/Tv Ratio	2.09 (0.05)	2.09 (0.03)	2.09 (0.03)	2.08 (0.04)	2.09 (0.07)	1.94	2.28

Quantitative data are expressed as medians and interquartile ranges or means and standard deviations. Coverage is expressed as depth of coverage (x). Proportions of observations covered at 20x, 10x and 0x are expressed as percentages (%). Proportions of observations with a Phred quality score of Q20 are expressed as percentages (%). Library sizes are presented in gigabases (billion base pairs). TiTv ratios are expressed as means and standard deviations.

### KAPA QC scores and coverage

**Fig 2:**
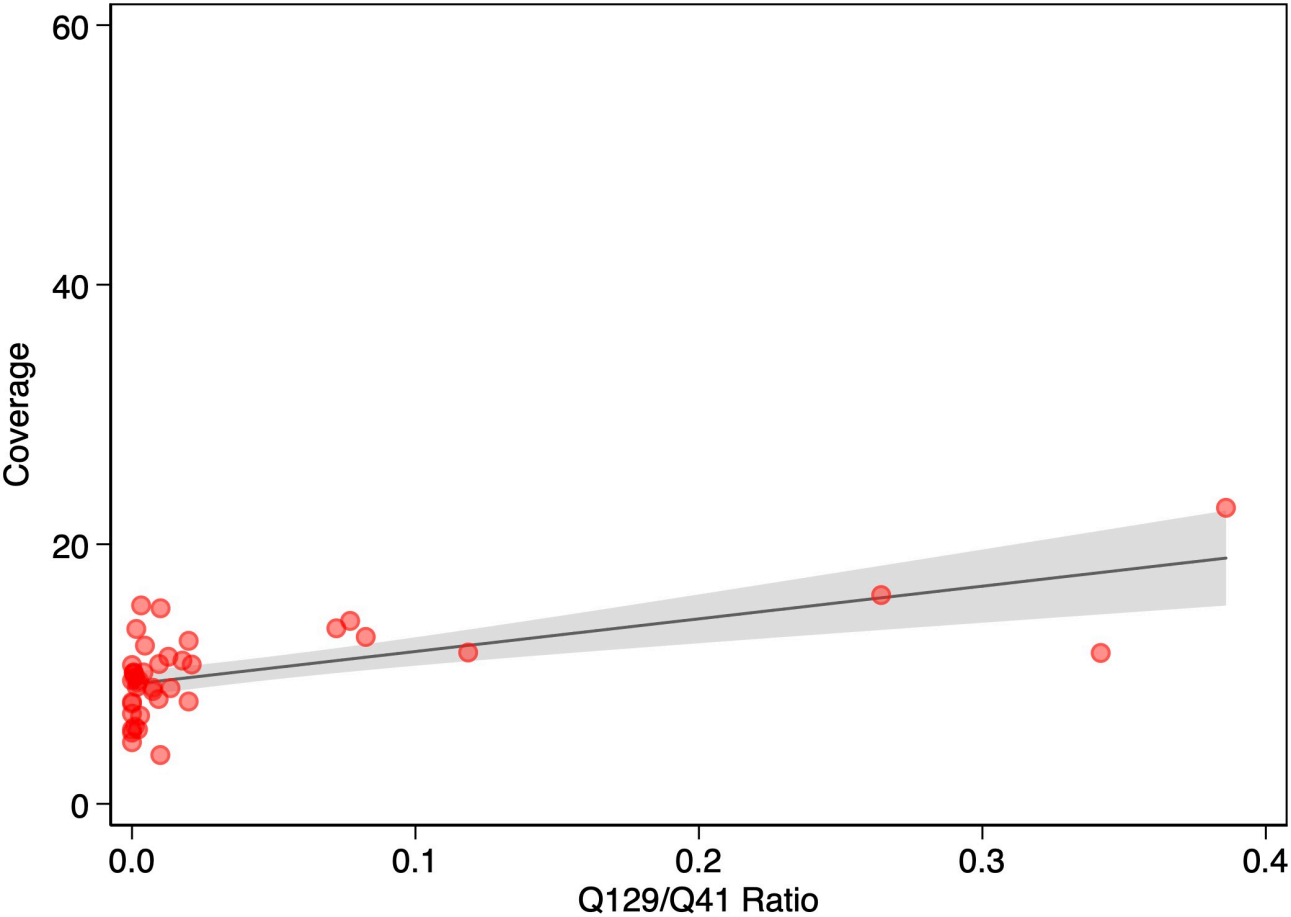
Association between Q129/Q41 ratio and coverage (n = 41). The solid line represents the linear prediction. The shaded area represents the 95% confidence interval for the linear prediction.

The median KAPA QC score (Q129/Q41 ratio, n = 41) for the WGS samples was 0.004 (IQR 0.0007–0.0178). The KAPA QC score was a good predictor of coverage (*P* = 0.000012). (Fig 2). Also, KAPA QC score was an independent predictor of coverage (*P* = 0.000039) in the multivariable regression analysis. In the same analysis, neither DNA yield (*P* = 0.584) nor storage time (*P* = 0.254) were predictors of coverage.

### Effects of storage time

**Fig 3:**
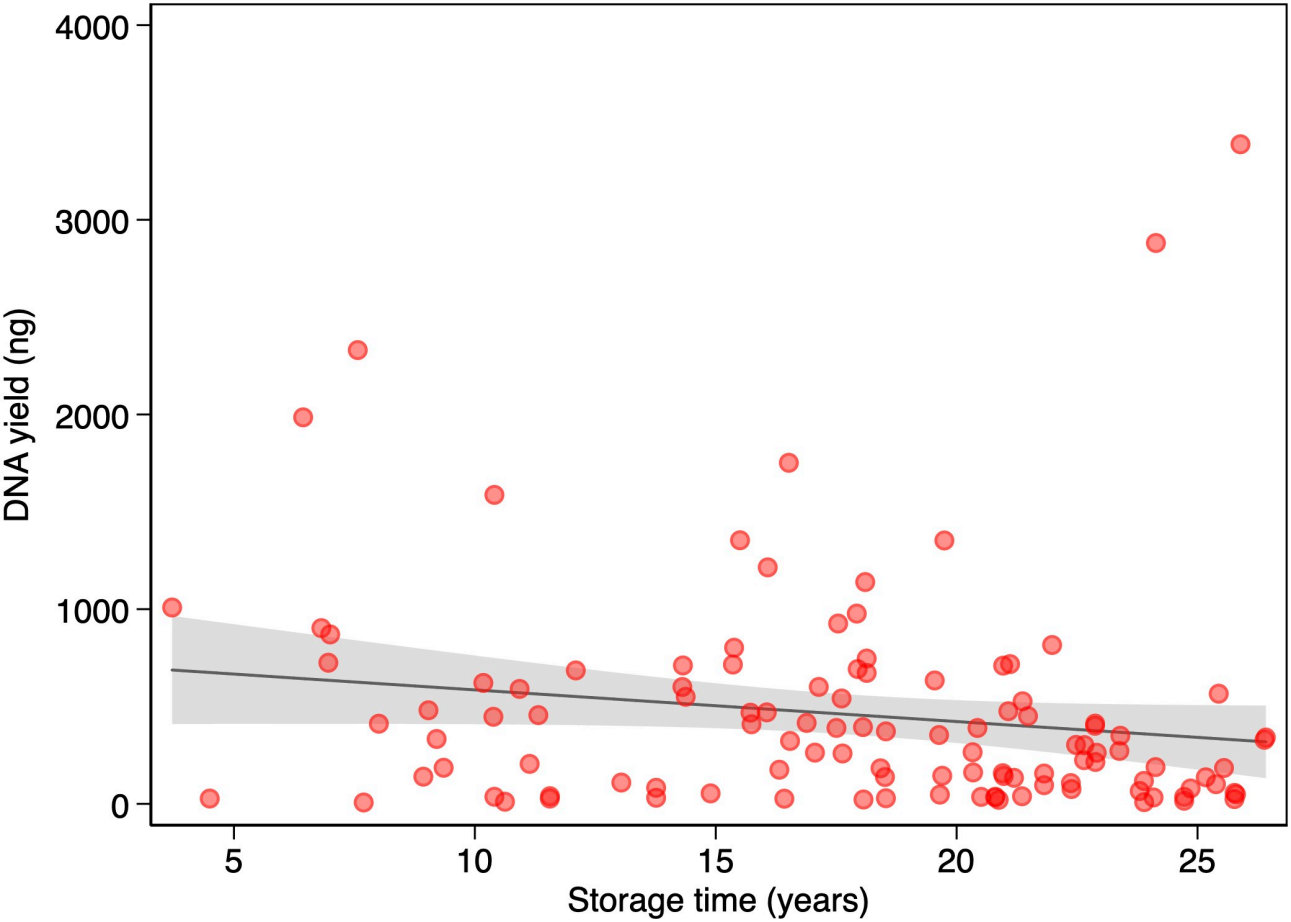
Association between storage time and DNA yield (n = 114). The solid line represents the linear prediction. The shaded area represents the 95% confidence interval for the linear prediction.

**Fig 4:**
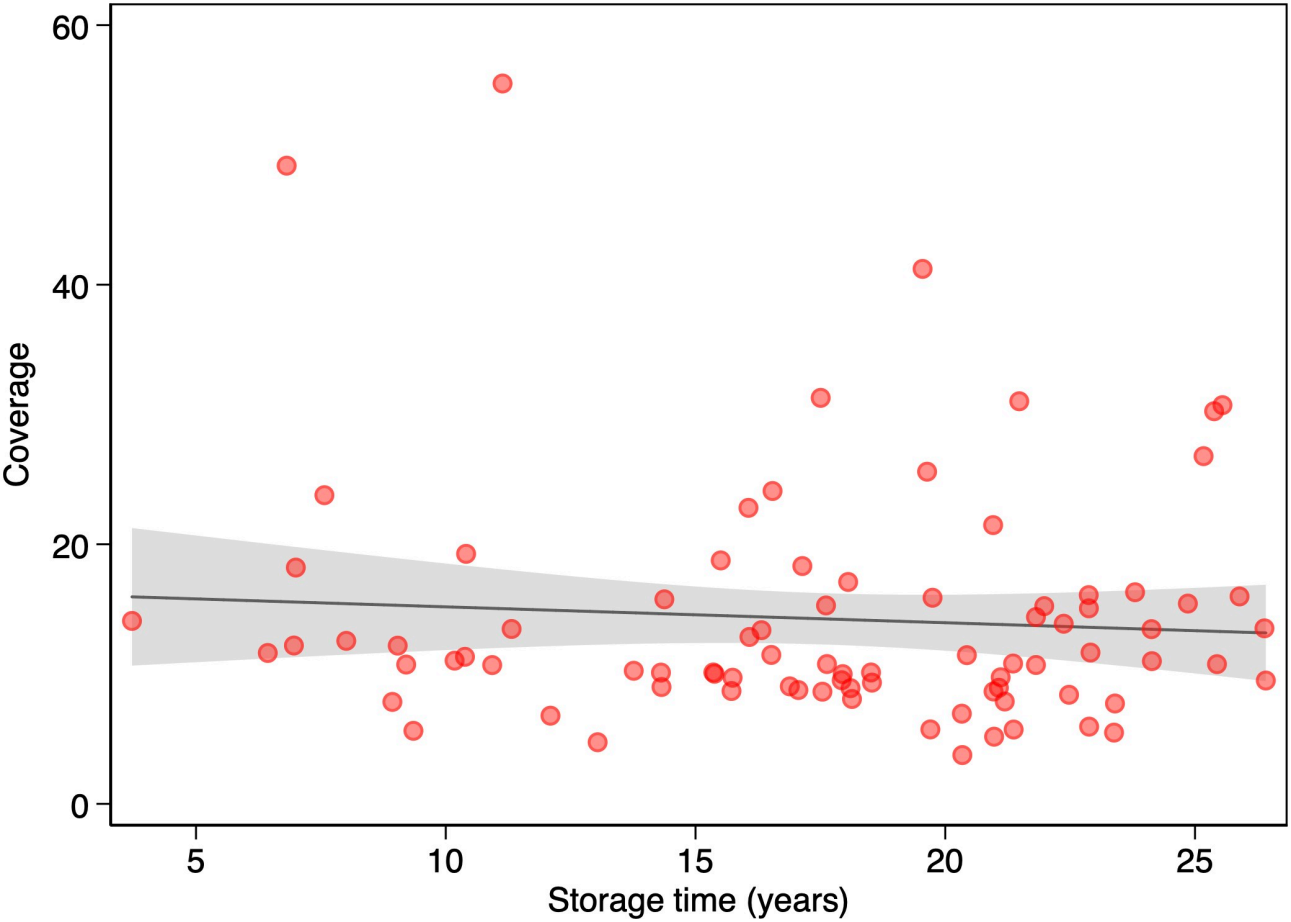
Association between storage time and coverage (n = 85). The solid line represents the linear prediction. The shaded area represents the 95% confidence interval for the linear prediction.

**Fig 5:**
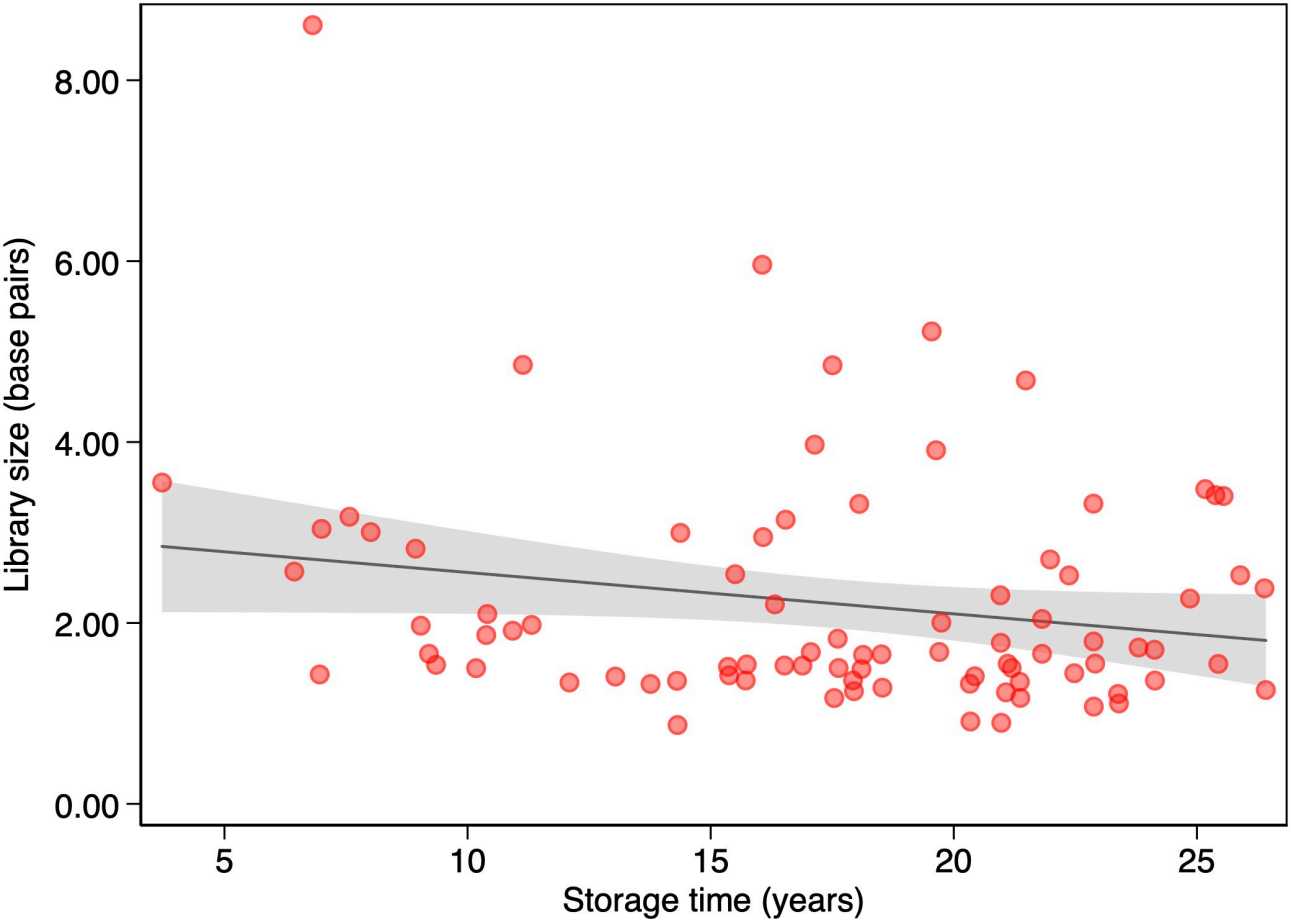
Association between storage time and library size (n = 85). The solid line represents the linear prediction. The shaded area represents the 95% confidence interval for the linear prediction.

Table 1 shows DNA yields for all samples and for samples grouped by 5-year or 10-year storage intervals. We found no association between DNA yield and storage time (*P* = 0.08) (Fig 3). Table 2 shows WGS metrics for all samples and for samples grouped by 5-year or 10-year storage intervals. We found no association between coverage and storage time (*P* = 0.495) (Fig 4). There were no apparent statistically significant differences in storage time according to linear regression (including log-transformed variables) or among storage groups according to the two-sided *t*-test or the Mann-Whitney *U* test. We found no association between library size and storage time (*P* = 0.065) (Fig 5) or between coverage and DNA yield (*P* = 0.671). There were no associations among coverage, storage time (*P* = 0.539), and DNA yield (*P* = 0.757) according to multivariable regression analysis.

## Discussion

This first of its kind study investigates the practicality of WGS analysis and the effects of storage time on formalin-fixed paraffin-embedded endomyocardial biopsies obtained from heart transplant recipients. DNA yield, coverage, and library size did not significantly decrease with FFPE storage time even up to 26 years. Previous studies have investigated the practicality of applying NGS to FFPE samples [4–9]. To the best of our knowledge, the present study is the first to test the feasibility of WGS in biopsies derived from heart transplant recipients.

Appropriate coverage is a prerequisite for accurate variant calling in human genome studies. However, the results of studies estimating the coverage required for accurate variant calling are highly variable. Ajay et al. showed that 95% of the genome was callable at 35x coverage. They also demonstrated that the increase in the proportion of callable genome rapidly declines at coverages > 25x. Therefore, increasing coverage above this level does not necessarily improve variant calling. Nevertheless, a coverage of 50x was recommended to call all single nucleotide polymorphisms (SNPs) and indels [10]. A more recent study on WGS coverage requirements used a different variant calling approach and determined that a mean coverage of 14x was necessary to call 95% of the SNPs accurately [11]. In the present study, many samples did not meet the 30x target coverage but nonetheless yielded sufficient coverage for variant analysis. Numerous samples can be sequenced from one biopsy. Moreover, the cost of WGS has markedly decreased. Therefore, sequencing can be repeated when the coverage is inadequate. In practice, coverage requirements depend on the objective of the analysis. Retrospective studies require less accuracy than diagnostic investigations.

Several samples yielded disappointing Q129/Q41 ratios from the KAPA QC assay (Q129/Q41 ratio). Nevertheless, Q129/Q41 may still predict coverage and many samples with poor Q129/Q41 ratios still yielded sufficient coverage. According to the present study, Q129/Q41 is not recommended as a WGS gatekeeper.

Detailed characterization of the long-term impact of variations in HLA and other genomic regions on outcomes requires sequencing and variant calling of FFPE samples stored for > 10 years. Potential applications of WGS on serial donor- and recipient samples include donor-recipient match/mismatch analysis, HLA interaction analysis and GWAS (genomewide association studies) including functional effect prediction analysis. Studies on the impact of FFPE storage time on NGS quality metrics reported decreases in library size and coverage with storage time (4). In the present study, WGS quality metrics decreased with increasing storage time but these changes were not statistically significant either by comparison between storage groups or by linear regression. However, statistical significance might have been detected if a larger number of biopsies been sampled. In any case, the WGS quality metrics for many older samples were adequate.

The TiTv ratios reported in the present study suggested that there were no artificial variants introducing bias. The TiTv ratios were within the expected range and were comparable to the reference sample NA12878. Therefore, the endomyocardial samples stored as FFPE were not genetically altered by FFPE processing [12].

Immune cell differentiation and function may depend on epigenetic mechanisms. Nevertheless, the influences of DNA methylation profiles on transplant acceptance, transplant rejection, and other post-transplant complications are unknown. The candidate genes contributing to this phenomenon can be identified by NGS and gene expression profiling. It is known that FFPE is suitable for use in DNA methylation analysis [13, 14]. DNA methylation analysis also has clinical potential in the early diagnosis of post-transplant complications [15]. Patients may be segregated into low-, intermediate-, and high rejection risk groups. New diagnostic and therapeutic methods could be developed by identifying genomic variants and aberrant DNA methylation. Moreover, immunosuppressive therapy can be adapted by using non-invasive blood sampling to isolate and quantify DNA methylation. Like other NGS studies, most DNA methylation studies are performed on fresh frozen tissue. Studies have shown comparable results when comparing NGS on fresh frozen samples to NGS on FFPE samples [16, 17]. Likewise, a study comparing DNA methylation using targeted sequencing on matched fresh frozen tissue and FFPE tissue found no effect on methylation calling and concordant results for several loci [18]. In this study we demonstrate the practicality of WGS on stored EMB samples. We therefore hypothesize that DNA extracted from EMB samples can be utilized in methylation analysis using NGS approaches and suggest that this be investigated further.

A limitation of the present study was the lack of sequencing techniques for comparison. The TiTv ratios calculated in this study were based on data from chromosome 6 alone because variant calling is very time-consuming. However, this limitation was partially offset by comparing the results for chromosome 6 to a reference genome. A strength of the present study was the ability to examine cardiac biopsies after heart transplantation, especially since all patients were transplanted at Skane University Hospital and all biopsies were processed by the same pathology unit.

## Conclusion

This study demonstrated the feasibility of whole-genome sequencing on formalin-fixed paraffin-embedded endomyocardial biopsies and indicated that this process can facilitate large-scale studies based on stored histopathological samples.

## Materials and methods

### Ethics statement

The study protocol was approved by the Regional Ethical Committee in Lund, Sweden (Nos. 2012/824, 2013/263, and 2016/112). The data were anonymized and de-identified prior to analysis.

### Study population

The study involved recipients who had undergone heart transplantation at Skane University Hospital, Lund, Sweden between 1988 and 2009. EMB samples were obtained from routine rejection monitoring and stored as FFPE samples. FFPE tissue samples were acquired before transplant (recipient heart), shortly after transplant (donor heart), and around the time of death (donor heart). If the DNA yield was inadequate, multiple fragments were taken from different FFPE tissue samples at the same time points and analyzed as a single sample. All patients transplanted at the center were followed up, according to routine protocols for post-transplant care with at least yearly controls until the time of death.

### FFPE fixation and storage process

Fixation, dehydration, and embedding were performed at the Department of Pathology, Skane University Hospital, Lund, Sweden. Tissues were fixed in 4% formaldehyde, dehydrated with Tissue-Tek Xpress x120 (Sakura Finetek Co. Ltd., Tokyo, Japan), and manually embedded in Histowax (Histolab Products AB, Gothenburg, Sweden) using a Tissue-Tek TEC 5 Tissue Embedding Console System (Sakura Finetek Co. Ltd., Tokyo, Japan). Fixation times varied with tissue size. Processing, fixation, dehydration, and embedding of older samples (1988–1999) involved a similar protocol. After processing, FFPE samples were stored at room temperature.

### DNA extraction, quantity and quality assessment

Samples were sent to the Broad Institute of MIT and Harvard, Cambridge, MA, USA for DNA extraction and WGS. The DNA was extracted from the FFPE samples as cores, scrolls, or slide sections. DNA extraction was conducted on all samples with a column-based QIAamp DNA FFPE Tissue kit (Qiagen, Hilden, Germany). After deparaffinization and de-crosslinking, the samples were lysed with proteinase. Buffering conditions were adjusted for optimal DNA binding to the column. Lysed samples were added to the column and the DNA was selectively bound to the column membrane as a contaminant and passed through enzyme inhibitors in the washing steps. The DNA was eluted with Tris-EDTA (TE) buffer, quantified in triplicate with the Quant-iT PicoGreen DNA assay kit (Thermo Fisher Scientific, Waltham, MA, USA), and normalized to a minimum concentration of 2 ng/uL. Aliquots of ≥ 150 ng per sample were transferred for library preparation using the one-well protocol developed by the Broad Institute [19]. One library was provided and the typical median library insert size was 330 bp. Samples did not undergo post shearing clean-up because they were already highly degraded. The Kapa QC Q-ratios for Q129/Q41 base-pair target regions were calculated with a Kapa Library Quantification and QC kit (Roche Diagnostics, Risch-Rotkreuz, Switzerland) [20]. Samples lacking accurately calculated storage times were excluded.

### Whole-genome sequencing

WGS was conducted on samples using the Illumina HiSeq X and Illumina HiSeq 2000 (Illumina, San Diego, CA, USA). It generated 150-bp paired-end reads and short-insert, paired-end reads (2 × 100 bp), respectively, to reach the 30x coverage target (where x = number of sequenced bases / total genome size). Binary Alignment Map (BAM) files were demultiplexed, aggregated, and aligned. All reads per sample with the same index were demultiplexed from the other samples in the same run. The samples were aggregated into one BAM file and aligned to the reference genome (GRCh37/Hg19). Recalibration, indel realignment, and duplicate marking were performed on the raw reads in the BAM files. The Picard Informatics Pipeline was used to aggregate all of the data from a particular sample into a single BAM file including all reads, all bases from all reads, and original- or vendor-assigned quality scores.

The BAM files were indexed with SAMtools [21]. GATK v. 3.5.0 (Broad Institute, Cambridge, MA, USA) was used for additional WGS sequencing quality metrics analyses and variant calling. *GATK-HaplotypeCaller* and *GATK-GenotypeGVCF* were used for variant calling. Joint genotype calling of single-nucleotide variants (SNVs) and indels (insertion or deletion) was performed according to GATK Best Practices [22–24]. Variant calling was run on each sample individually and Genomic Variant Calling Format (GVCF) files were generated. The GVCF files were combined into one Variant Call Format (VCF) file. All 85 samples were included in joint genotyping processing. *GATK-VariantRecalibrator* and *GATK-ApplyRecalibration* were used for variant filtering. *GATK-SelectVariants* was used to isolate individual samples which did not pass variant filtering. Additional and complementary sequencing quality metrics and coverage analyses were performed with *GATK-CollectWgsMetrics* and *GATK-VariantEval*. *GATK-CollectWgsMetrics* calculated the mean coverage using overlap and duplication values [25]. These analyses generated data on coverage, TiTv ratios, and the mean percentages of the genome at 20x, 10x, and 0x coverage. Phred scores were available from the original- or vendor-assigned quality scores. A Phred score of Q20 indicated a 1:100 chance of an incorrect base call and was used during variant filtering [26]. The TiTv ratio is the ratio of transitions (point mutation between purines A ↔ G or pyrimidines C ↔ T) to transversions (point mutations between different types of nucleotides: A ↔ C, A ↔ T, C ↔ G or G ↔ T) in the SNVs in a WGS file. Since there are more possible transversions, the theoretical ratio is 0.5. The most common mutation is a cytosine (C) to thymine (T) transition. The expected whole-genome sequencing TiTv ratio in humans is therefore 2.0–2.1 [27]. TiTv is relatively higher in exonic regions and comparatively lower in intronic regions. Only WGS data for chromosome 6 was used in this study. Storage times were calculated for all WGS samples.

### Statistical analysis

Data are presented as means ± SD or medians and interquartile ranges (IQR). Categorical variables are percentages. WGS quality metrics were compared between groups by a two-sample *t*-test and by a Mann-Whitney *U* test for continuous variables. The associations among WGS quality metrics, storage time, and KAPA QC scores were evaluated by linear regression (including log transformed variables). KAPA QC, DNA yield, and storage time were factored into the multivariable regression analysis of the WGS quality metrics. *P* < 0.05 was deemed statistically significant. Data were processed in Stata/IC v. 15.1 (StataCorp LP, College Station, TX, USA).

## Supporting information

S1 Table DNA and sequencing metrics for all samples. (XLSX)
